# Concerted Action by State Boards of Health

**Published:** 1884-02

**Authors:** 


### Society Reports.

---

### CONCERTED ACTION BY STATE BOARDS OF HEALTH.

[Reported for the CHICAGO MEDICAL JOURNAL AND EXAMINER.]

There has been a growing conviction among leading sanitarians intrusted with the official execution of practical health measures, that while the work of the American Public Health Association is of inestimable value in promoting the interests of sanitary science and sanitary reform, there is a constantly increasing need for an annual conference of State and other health officials in regard to practical affairs of their everyday work, some part of which work cannot profitably be discussed in a public meeting consisting largely of persons not familiar with its details.

After due consideration, a meeting of representatives of State Boards was held at Detroit during the recent meeting of the American Public Health Association, at which, after discussion, it was decided to call a meeting of the secretaries or other representatives of all State Boards of Health, in Washington, during May, 1884, for the purposes mentioned, and with the view of organizing a section devoted to State Board work in the present Association, or the formation of a permanent separate organization especially adapted to the needs of State Boards of Health. Drs. Henry B. Baker, of Michigan, and J. N. McCormack, of Kentucky, were appointed a committee to confer with and secure the coöperation of all the State Boards in fulfilling the object of the meeting, and Drs. C. W. Chamberlain, of Connecticut, J. E. Reeves, of West Virginia, and Stephen Smith, of New York, were appointed a committee on organization, to report at the meeting in May. The American Medical Association meets in Washington in May ; and another reason for holding the meeting in Washington, is that the representatives of the State Boards may also have an opportunity for conferring with the Senators and Representatives in Congress from their respective States, in regard to national sanitary legislation. It would seem that whenever the health authorities of all the States shall meet, discuss, and agree upon the course they will pursue with respect to yellow fever, cholera, small-pox, or any disease which endangers public health without regard to State lines or borders, and whenever all State Boards shall act in concert, considerable progress will have been made in solving the problem of what are the best methods for national action in regard to inter-State and maritime quarantine, or inspection and disinfection, as well as in the practical control of epidemic diseases within the several States of this country.

